# Empirical Comparison of the Breslow Estimator and the Kalbfleisch Prentice Estimator for Survival Functions

**DOI:** 10.4172/2155-6180.1000392

**Published:** 2018-02-28

**Authors:** Fang Xia, Jing Ning, Xuelin Huang

**Affiliations:** Department of Biostatistics, The University of Texas MD Anderson Cancer Center, Houston, TX 77030, USA

**Keywords:** Breslow estimator, Kalbfleisch prentice estimator, Survival analysis

## Abstract

When analyzing time-to-event data in a non-parametric setting without considering covariates, the Kaplan-Meier estimator is widely used to estimate the survival function. When considering covariates, the Cox proportional hazards model is widely used to account for covariates effects. In this setting, for the baseline survival function, the most commonly used approach is the Breslow method, which estimates the baseline survival function as an exponential function of the cumulative baseline hazard function. However, an unnatural and undesirable feature of the Breslow estimator is that, its estimated survival probability will never reaches zero even if the last observation is an event. In this article, we consider an less commonly used alternative, the Kalbfleisch Prentice estimator for the baseline survival function. It is the counterpart of the Kaplan-Meier estimator in a setting with covariates, and thus similarly as the Kaplan Meier estimator, it will reach zero if the last observation is an event. To evaluate the usefulness of the Kalbfleisch Prentice estimator and its relative performance comparing with the Breslow estimator, we conduct simulation studies across a range of conditions by varying the true survival time distribution, sample size, censoring rate and covariate values. We compare the performance of the two estimators regarding bias, mean squared error and relative mean squared error. In most situations in our study, the Kalbfleisch Prentice estimator results in less bias and smaller mean squared error than the Breslow estimator. Their differences are especially clear at the tail of the distribution. The implications of such differences in applications are discussed. We advocate the use of Kalbfleisch Prentice estimator in practice, and further research on its properties.

## Introduction

Survival analysis is commonly used in biomedical sciences to analyze time-to-event outcome data. The Kaplan-Meier (KM) estimator [1] is often considered the “golden standard” for estimating a survival distribution without covariates. It is a piecewise continuous function and can be regarded as a point estimate of the survival function at a given time. An alternative is the Nelson-Aalen (NA) estimator [2,3], which is based on the exponential function of the cumulative hazard function. Fleming and Harrington proved that the KM estimator and the NA estimator are asymptotically equivalent [4]. Anderson et al. [5] and Fleming and Harrington [4] derived their asymptotic properties using a counting process. Bohoris addressed the finding that the NA estimator is consistently larger than the KM estimator [6]. Klein, Moeschberger [7,8] and Colosimo et al. [9] showed that the NA estimator outperforms the KM estimator for small sample sizes.

In estimating a survival distribution with covariates, Cox proposed a proportional hazards model that specifies the conditional hazard function of the failure time for a given set of covariates [10]. The Cox proportional hazards (PH) model assumes that the hazard function at time *t* for a given covariate vector *Z,Z*=*(Z_1_, …, Z_q_)^T^* is the product of an arbitrary baseline hazard function and an exponential function of the linear combination of the covariates. It can be written as

(1) $$\lambda (t|Z)=\lambda_{0}(t)\;\exp(\beta^{T}Z),$$

where *λ*_0_(*t*) is the baseline hazard function. The baseline hazard function is left unspecified, thus the Cox PH model is semiparametric.

For a random patient with covariates $Z=z^{*}={\left(z_{1}^{*},\ldots ,z_{q}^{*}\right)}^{T}$, the survival distribution can be estimated as eqn. (2):

(2) $$\hat{S}(t|Z=z^{*})={\hat{S}}_{0}(t{)}^{\exp({\hat{\beta }}^{T}z^{*})}$$

where ${\hat{S}}_{0}(t)$ is the estimated baseline survival function and *β*^ is the regression coefficients that can be estimated using the partial likelihood without specifying the baseline hazard function [10,11].

To estimate the baseline survival function *S*_0_(t), the Breslow estimator [12] or the Kalbfleisch Prentice (KP) estimator [13,14] can be used. The Breslow estimator uses the profile likelihood approach by extending the NA estimator. The KP estimator uses the discrete failure time to approach a continuous function, which is analogous to the KM estimator. For time-to-event data, it is desirable for the estimated survival probability to reach zero if the last observation is an event. Due to the exponential part of the Breslow estimator, the estimates will always be positive. The KP estimator will reach zero if the last observation is an event, but it requires more complex computations.

More asymptotic properties of the Breslow estimator have been studied than that of the KP estimator. Tsiatis [15] and Anderson et al. [5] derived the asymptotic distribution of the Breslow estimator. Without considering covariates or the Cox PH model, Huang and Strawderman established new formulas for the bias and the mean squared error (MSE) of the Breslow estimator using the It ô stochastic integration formula for semimartingales [16]. When the covariates are accounted for by the Cox PH model, the relative performance of the Breslow estimator and the KP estimator is not clear. More empirical results to compare the relative performance of these estimators are still necessary.

In this article, we focus on the empirical comparison of the Breslow estimator and the KP estimator regrading bias, MSE and relative MSE (RMSE). Section 2 reviews the Breslow estimator and the KP estimator. Section 3 presents a comprehensive simulation study to examine the relative performance of these estimators under settings of various baseline survival functions, sample sizes, censoring rates and covariate values settings.

## Method

In this section, we review the methodology of the Breslow estimator and the KP estimator for survival functions. For each patient *i*, let *T_i_* denote the time to the event of interest and *C_i_* denote the censoring time, *i*=1, …, *n*. Then the observed data are *X_i_*=*min*(*T_i_*,*C_i_*) and the censoring status is Δ_*i*_=*I*[*Ti* ≤ *Ci*]: Δ_*i*_=1 if an event was observed for the *i*th patient; Δ_*i*_=0 if patient *i* was censored. We use the upper case to denote random variables and the lower case to denote realized values.

### The Breslow estimator

Let R(*xj*) be the set of patients who are still at risk of experiencing the event at time *xj*. For patient *i*, whose covariate vector is *zi*=(*zi1*, …, *ziq*)^*T*^, Breslow [12] proposed a nonparametric maximum likelihood estimator for the cumulative baseline hazard function ${\hat{\Lambda }}_{0,\mathrm{BR}}(t)$, which in the situation of no ties between the observed event times can be written as:

Λ^0,BR(t)=∑j:xj≤t{δjΣk∈R(xj)exp(β^TZk)}

Then the Breslow estimator for the baseline survival function is:

$${\hat{S}}_{0,\mathrm{BR}}(t)=\exp\{-{\hat{\Lambda }}_{0,\mathrm{BR}}(t)\}$$

Thus, the Breslow survival function estimator for a subject with covariate vector *Z*=z* can be derived as:

$${\hat{S}}_{\mathrm{BR}}(t|Z=Z^{*})={\left[{\hat{S}}_{0,\mathrm{BR}}(t)\right]}^{\exp(\hat{\;}z)}=e^{\exp(\hat{\;}Tz^{*})}{\hat{\Lambda }}_{0,\mathrm{BR}}|(t)$$

### The Kalbfleisch-Prentice estimator

The later proposed Kalbfleisch Prentice (KP) estimator [13,14] for survival functions uses the discrete failure time to approach a continuous function. Let *D*(*xj*) be the set of patients who failed at time *xj*. For distinct failure time xj, j1, …, m,

$$x_{1}<x_{2}<\ldots <x_{m}.$$

Let *α_j_* denote the conditional probability of surviving at time *x_j_* for a subject at baseline. The baseline survival function can be estimated as

$${\hat{S}}_{0,\mathrm{KP}}(t)=\prod_{j:x_{j}\leq t}{\hat{\alpha }}_{j}^{\delta_{j}}$$

This leads to the likelihood function *L*:

$$L=\prod_{j=1}^{m}\prod_{l\in D(x_{j})}(1-\alpha_{j}^{\exp(\beta^{T}z_{l}^{*}})\prod_{k\in R(x_{j})-D(x_{j})}\alpha_{j}^{\exp({\hat{\beta }}^{T}z_{k}^{*})}$$

Take the estimated $\beta^{T}={\hat{\beta }}^{T}$ from the partial likelihood and maximize the likelihood function *L* with regard to *αj*, which is the solution of the equation

$$\sum_{l\in D(x_{j})}\frac{\exp({\hat{\beta }}^{T}Z_{l})}{\left(1-\alpha_{j}^{\exp(\beta^{T}z_{l}^{*}}\right)}=\sum_{K\in R(x_{j})}\exp({\hat{\beta }}^{T}Z_{k})$$

where zj is the set of covariates for a subject who died at xj. Assuming no ties, the solution α^j is

$${\hat{\alpha }}_{j}=[1-\frac{\exp({\hat{\beta }}^{T}Z_{j})}{\Sigma_{k\in R(x,j)}\exp({\hat{\beta }}^{T}Z_{k})}]\exp(-{\hat{\beta }}^{T}z_{j})$$

Accordingly, the KP estimated survival function for a subject with covariates *Z*=z* is:

$${\hat{S}}_{\mathrm{KP}}(t|Z=z^{*})={\left[{\hat{S}}_{0,\mathrm{KP}}(t)\right]}^{\exp({\hat{\beta }}^{T}z^{*})}={\left[\prod_{j:x_{j}\leq t}{\hat{\alpha }}_{j}\right]}^{\exp({\hat{\beta }}^{T}z^{*})}$$

## Simulations

**Figure 1:**
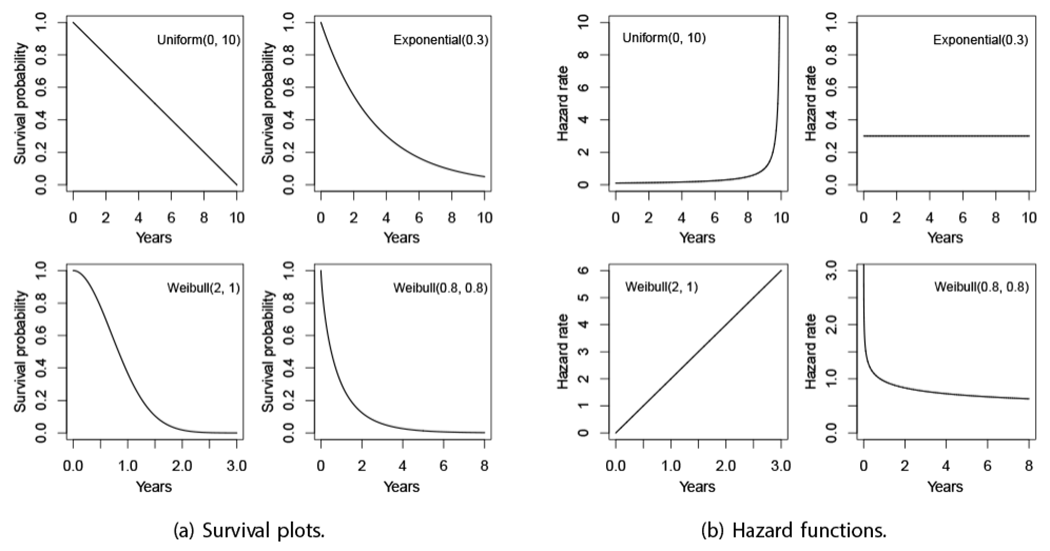
The survival plots and hazard functions of the four baseline survival distributions, (a) Survival plots, (b) Hazard functions.

In this section, we present four scenarios under which to evaluate the relative performance of the Breslow estimator and the KP estimator. We consider four baseline survival distributions: uniform, exponential, and two Weibull distributions. For the Weibull distributions, we include both cases of increasing and decreasing hazard functions. The baseline survival function of the Weibull distribution is given as:

S(t)=exp{−(t∕b)a},t>0,

where *a* is the shape parameter and *b* is the scale parameter. The baseline survival plots and hazard functions of all four scenarios are presented in Figure 1.

The survival time is generated using the Cox PH model in equation (1) with one covariate *z, z* ~ *Unif* (0, 10), and the regression coefficient *β* is set to be 0.2. The censoring time *C* is generated from either a uniform distribution or a exponential distribution. The distribution of censoring time *C* is deliberately calibrated to obtain the desired censoring rate *r*. For each example baseline survival distribution, we vary the sample size, censoring rate and covariate values, and compare the performance according to the bias, MSE and RMSE. Let *S*(*t*|*Z*=z*) denote the true survival function for a subject with covariate vector Z=z* and *G* denote the number of simulations. The bias and MSE of the Breslow estimators are calculated via simulation as follows:

$$\begin{array}{cc} {\text{Bias}}_{\left(Z=z^{*},\mathrm{BR}\right)}= & \frac{1}{G}\overset{G}{\underset{m=1}{\Sigma }}{\hat{S}}_{\mathrm{BR},m}(t|Z=z^{*})-S(t|Z=z^{*})\\ {\mathrm{MSE}}_{\left(Z=z^{*},\mathrm{BR}\right)}= & \frac{1}{G}\overset{G}{\underset{m=1}{\Sigma }}{\left[{\hat{S}}_{\mathrm{BR},m}(t|Z=z^{*})-S(t|Z=z^{*})\right]}^{2} \end{array}$$

The bias and MSE of the KP survival function estimator for the given covariate vector *Z*=z* can be computed similarly.

The RMSE is calculated as

$$\mathrm{RMSE}=\frac{{\mathrm{MSE}}_{\left(Z=z^{*},\mathrm{BR}\right)}}{{\mathrm{MSE}}_{\left(Z=z^{*},\mathrm{KP}\right)}}$$

The simulation settings used to study the effect of sample size, the censoring rate and covariate values are listed in Table 1. For the effect of the sample size, we let *n*=20, 50, 100 and keep *r*=25% and z*=5. For the effect of the censoring rate *r*, we generate data without censoring (*r*=0) and calibrate the censoring time distribution to generate data with censoring rates *r*=25%, 45%. For the effect of the covariate value, we assume z*=0, 5, 10 and fix *n*=50 and *r*=25%. Under each scenario, 5000 simulations are generated.

### Results: Uniform distribution

**Figure 2:**
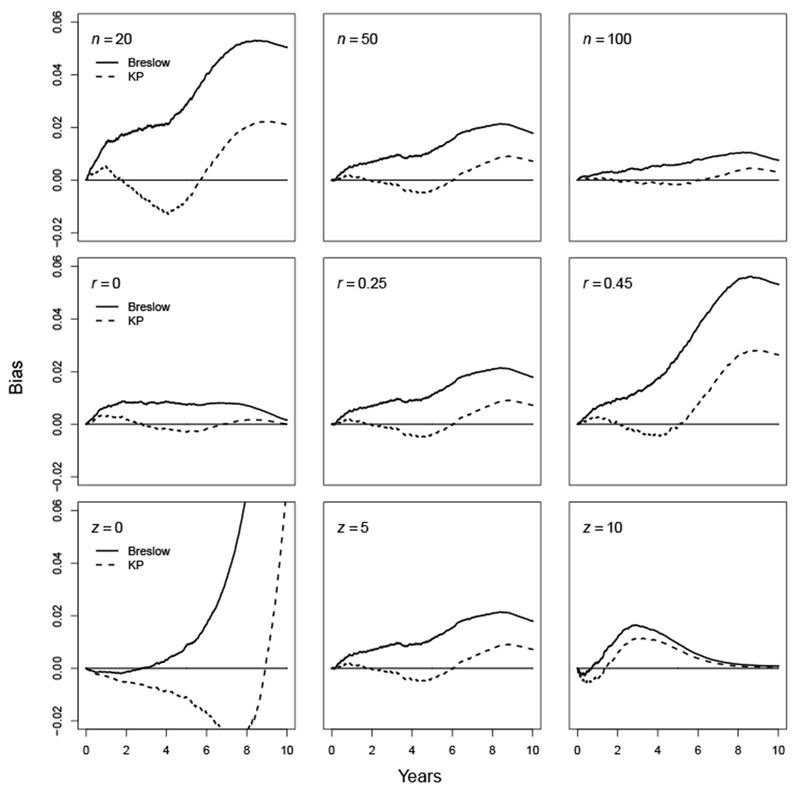
Bias for survival estimates of a uniform baseline survival distribution (0, 10).

Assuming a uniform distribution (0, 10) for the baseline survival function, the survival probability should reach 0 when time *t* equals 10. For this example, the magnitude of the bias decreases as the sample size *n* increases (Figure 2). The Breslow estimator always overestimates the survival probabilities, particularly for small sample sizes: the maximum bias is approximately 0.05 for *n*=20, 0.2 for *n*=50 and 0.01 for *n*=100. The KP estimator may overestimate or underestimate the survival probabilities. In particular, for *n*=20, the KP estimates are positively biased before *t*=2, then become negatively biased until *t*=6, and are positively biased again until *t*=10. The magnitude of the bias for the KP estimator is much smaller than that for the Breslow estimator. For the censoring rate *r*, the bias increases dramatically as *r* increases. The patterns are similar to the effect of the sample size. For the covariate value *z*, the bias decreases as *z* increases. For *z*=0, the bias of the Breslow estimates increases monotonically and reaches approximately 0.22 at *t*=10 (this part of the plot is truncated to keep the same range for all the y-axes). For *z*=5 and 10, the bias is less than 0.05. The Breslow estimator is still always positively biased, while the KP estimator underestimates the survival probabilities until *t*=8.5, then overestimates them until *t*=10. In all situations, the KP estimator has smaller bias than the Breslow estimator.

**Figure 3:**
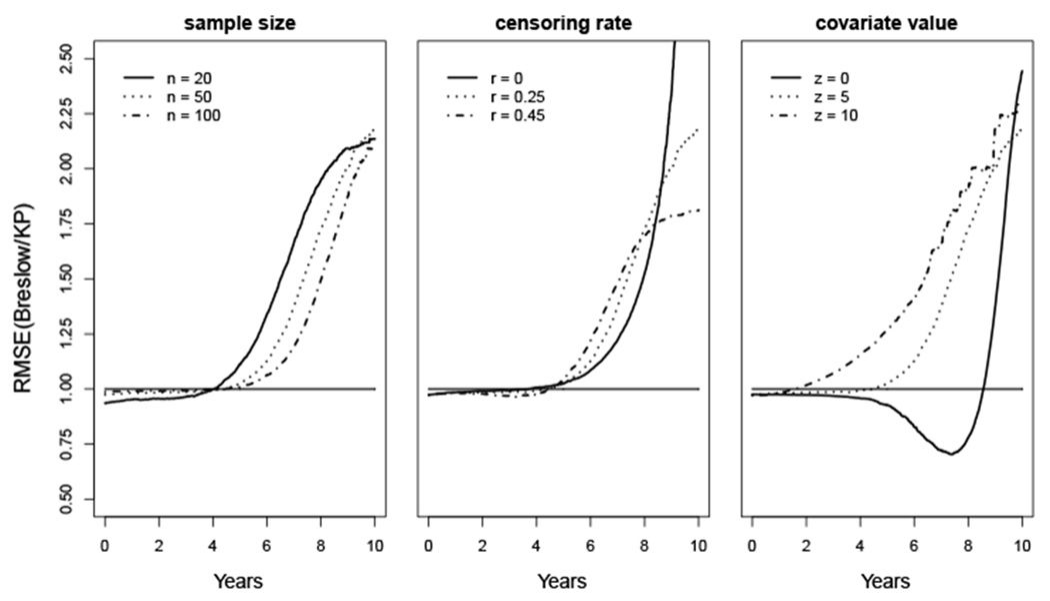
RMSE for survival estimate of a uniform baseline survival distribution over interval (0, 10).

Figure 3 presents the RMSE of the Breslow estimates compared to the KP estimates. For sample sizes *n*=20, 50 and 100, the RMSE stays slightly below 1 until approximately *t*=4, then substantially exceeds 1 and keeps increasing as time *t* increases. That is to say, compared to the KP estimator, the Breslow estimator has almost identical MSE for small values of *t*, but has substantially larger MSE for large values of *t*. Their differences increases when the sample size *n* decreases.

For the effect of the censoring rate *r*, the RMSE stays close to 1 before *t*=4, and then increases dramatically. In particular, for *r*=0, the RMSE keeps increasing as the MSE of the KP estimator reaches 0 at the tail. This is because in the situation without censoring, when *S*(*t*)=0 for *t* ≥ 10, all the events happen at time *t* ≤ 10. Therefore, $\mathrm{MSE}({\hat{S}}_{\mathrm{KP}}(t))=0$ for *t* ≥ 10.

For the covariate value *z*=0, the RMSE starts slightly below 1, then decreases to about 0.7 until it reaches *t*=7.5, then keeps increasing and reaches 2.3 at *t*=10. For covariate values *z*=5 and 10, the RMSE is always greater than 0.98 and exceeds 2 at *t*=10.

Summarizing all the scenarios, the Breslow estimator has substantially larger MSE than the KP estimator when *t* is relatively large. When *t* is small, the differences between the MSE for these estimators are minimal.

### Results: Exponential distribution

**Figure 4:**
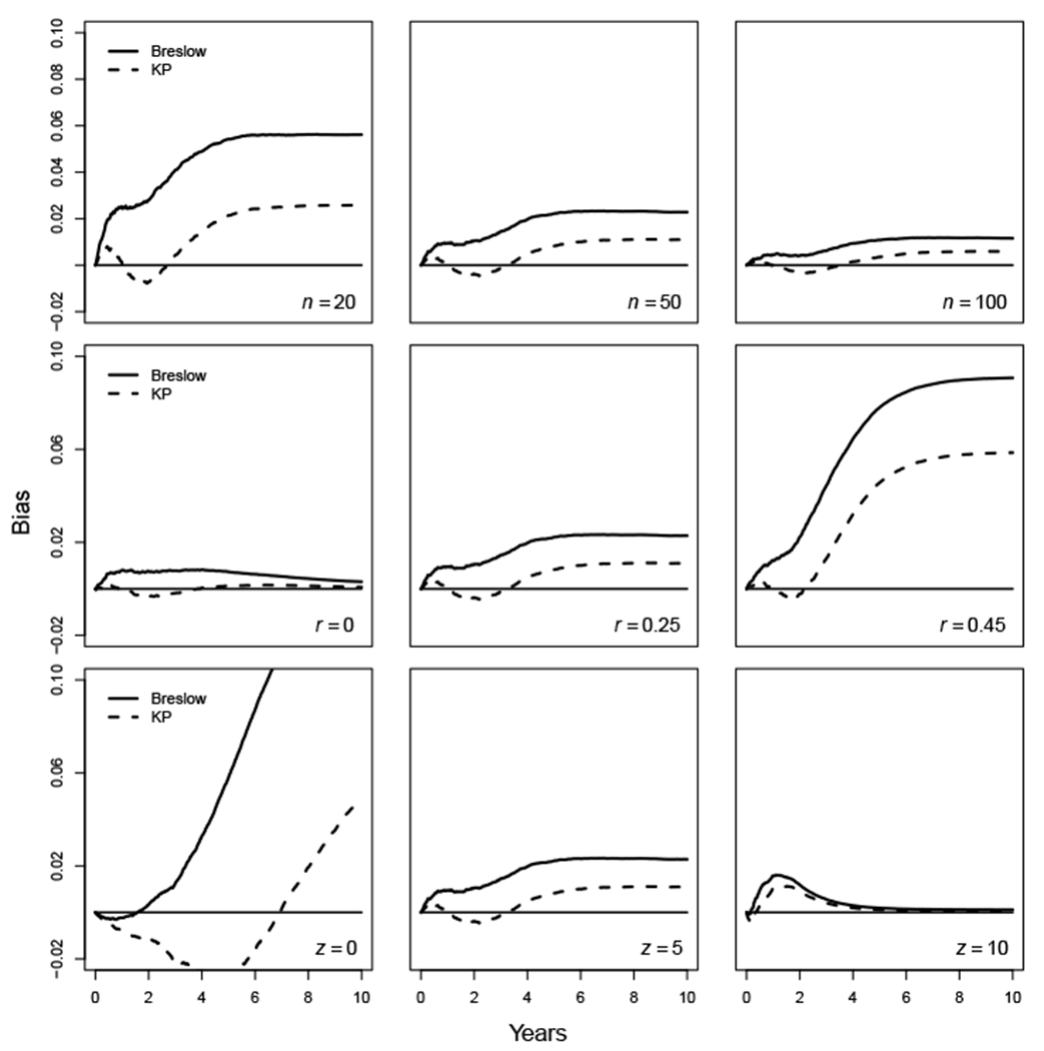
Bias for survival estimates of the exponential baseline survival distribution with λ=0.3.

Assuming the true baseline survival time follows an exponential distribution with the rate equal to 0.3, the baseline survival function *S*(*t*) at the end of the time line, *t*=10, is 0.091. The bias plots for the effect of sample size *n*, censoring rate *r* and covariate values *z* are presented in Figure 4. They display a pattern that is very similar to that obtained from the setting of the uniform baseline survival function. In general, bias decreases as *n* and *z* increase, or *r* decreases. The magnitude of the bias for the Breslow estimator is almost always larger than that for the KP estimator.

**Figure 5:**
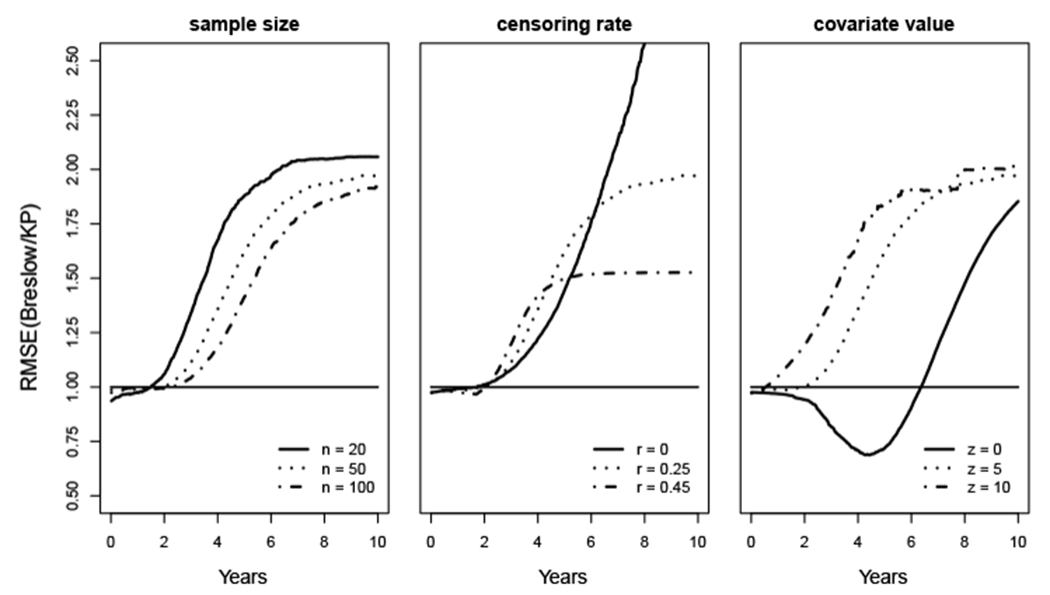
RMSE for survival estimates of an exponential baseline survival distribution with λ=0.3.

The RMSE for the exponential baseline survival function is shown in Figure 5. For sample size *n*=20, 50 or 100, the RMSE starts slightly below 1, then increases quickly and at *t*=10 reaches the maximum value, which is 2.15 for *n*=20, 1.9 for *n*=50, and 1.8 for *n*=100. For the scenario of *r*=0, the RMSE approaches infinity as *t* increases. This is because at *t*=10, the KP estimate *S*(*t*=10) is 0, and thus the *MSEKP* (*t*=10) is also 0. For *r*=0.45, the RMSE starts near 1, then keeps increasing and becomes steady at 1.5 after *t*=4. For the effect of covariate values *z*, the RMSE at baseline where *z*=0 is below 1 until *t*=6.2, with its minimum value approximately 0.76 at *t*=4.2, then keeps increasing and reaches 1.75 at *t*=10. Whereas for *z*=5 and 10, the RMSE keeps increasing and reaches near 2 at *t*=10. In most scenarios, the RMSE exceeds 1. This implies that the KP estimator has smaller MSE compared to the Breslow estimator.

### Results: two weibull distributions

For the Weibull distribution as the true baseline survival function, we consider two cases: a Weibull distribution with a shape parameter (*a*) equal to 2 and a scale parameter (*b*) equal to 1, and another Weibull distribution with *a*=*b*=0.8. The Weibull distribution with *a*=2 corresponds to an increasing hazard, and that with *a*=0.8 corresponds to a decreasing hazard. For Weibull (2,1), the true baseline survival function becomes sufficiently close to 0 (0.0001) at *t*=3. For Weibull (0.8,0.8), the true baseline survival function becomes close to 0 (0.0018) at *t*=8.

**Figure 6:**
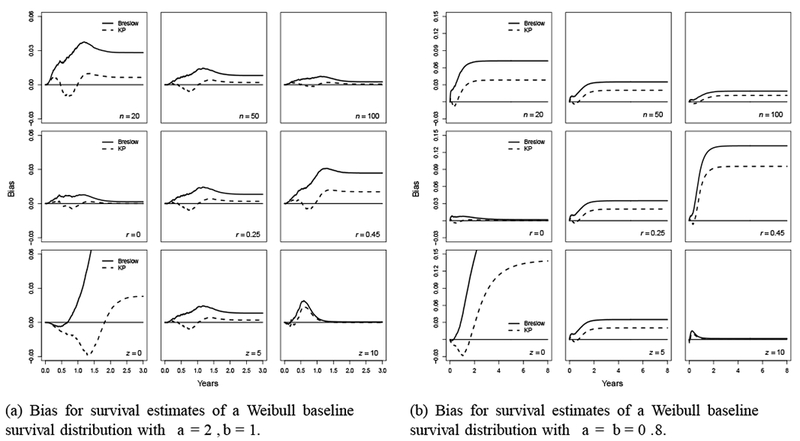
Bias for survival estimates of two Weibull distributions: (a) Bias for survival estimates of a Weibull baseline survival distribution with a=2, b=1; (b) Bias for survival estimates of a Weibull baseline survival distribution with a=b=0.8.

The bias plots for Weibull (2,1) are presented in Figure 6a. They display patterns similar to those for previous scenarios in smaller magnitude. For Weibull (0.8, 0.8), the bias decreases as *n* or *z* increases, or as *r* increases (Figure 6b). For all scenarios under Weibull (0.8, 0.8), the bias reaches a plateau after *t*=2. Generally, the magnitude of the Breslow estimator is larger than that of the KP estimator.

**Figure 7:**
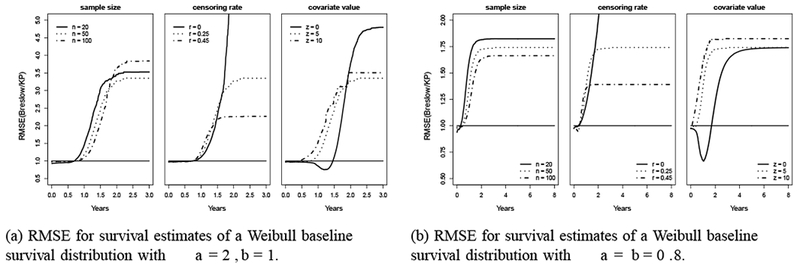
RMSE for survival estimates of two Weibull distributions: (a) RMSE for survival estimates of a Weibull baseline survival distribution with a=2, b=1; (b) RMSE for survival estimates of a Weibull baseline survival distribution with a=b=0.8.

The RMSEs for Weibull (2,1) and Weibull (0.8, 0.8) are presented in Figure 7a and 7b, respectively. For Weibull (2,1), the pattern of the RMSE is similar to the one with uniform (0,10) but in larger magnitude for RMSE values exceeding 1. For Weibull (0.8, 0.8), in most scenarios except for *r*=0 and *z*=0, the RMSE starts right below 1, then increases rapidly and reaches a plateau before *t*=2. For the scenario of *r*=0, the RMSE increases dramatically to a substantially larger value as the Sˆ for the KP estimator approaches 0. For the *z*=0 scenario, the RMSE stays below 1 until *t*=2, then keeps increasing and stays at 1.6 at *t*=5; whereas for *z*=5 or 10, the RMSE monotonically increases and stays around 1.75 since *t*=2. The RMSE results show that the MSE of the Breslow estimator is almost always larger than that of the KP estimator, except when *z*=0.

## Discussion

In this article, we compare the Breslow estimator and the KP estimator for survival functions. In most situations in our study, the KP estimator has smaller bias and MSE compared to the Breslow estimator. The bias, MSE and RMSE are influenced by the sample size, censoring rate and covariate values.

For survival analysis involving time-to-event data, it is desirable for the estimated survival probability to be zero at the tail of the distribution if the last observation is an event. The KP estimator satisfies this property, but the Breslow estimator does not. The Breslow estimator is in the form of an exponential function of the cumulative baseline hazards, which will always be strictly positive. On the other hand, the KP estimator is formed as a product-limit, similar to the KM estimator. It uses the discrete failure time approach to account for covariate effects through the Cox PH model. It will reach zero if the last observation is an event.

When the survival plot has a plateau at the tail, we may consider using the mixture cure model [17] for long-term survivors. The mixture cure model assumes the population is composed of a cured proportion and an uncured proportion. For the uncured proportion, the survival function should be zero when time goes to infinity. When checking the goodness-of-fit for cure models, it is important to have an accurate estimate of the survival probability at the tail. The Breslow estimator may not be a good choice as it will never reach zero due to its exponential form. Many researchers of mixture cure models experience convergence problems when they use the Breslow estimator for the survival function of the uncured subpopulation. A commonly used approach to fix this problem is to assign an arbitrarily large value on the last jump size at the tail of the cumulative hazard function so that the Breslow estimator will be sufficiently close to zero [18–20]. However, such a change increases the bias for estimates [21]. Viewing this problem, a solution may be to use the KP estimator to replace the Breslow estimator in the fitting of the mixture cure models. Our preliminary simulation studies show that the KP estimator provides a more accurate estimation of cure rates than the Breslow estimator. These results may be used to check the goodness-of-fit of cure rate models [22,23], which warrants further research.

## Figures and Tables

**Table 1:** Simulation settings.

	Variable of interest	Fixed parameters
Sample size	n=20, 50, 100	r=25%, z*=5
Censoring rate	r=0, 25%, 45%	n=50, z*=5
Covariate value	z=0, 5, 10	n=50, r=25%
